# Umbilical cord blood infusion enhances hematopoietic recovery and improves survival in elderly AML patients with chemotherapy-induced myelosuppression: a retrospective study

**DOI:** 10.3389/fonc.2026.1867070

**Published:** 2026-08-26

**Authors:** Yimin Zhou, Yuye Ren, Lan Zhang, Wenya Hu, Jiao Ma, Xiaomin Wang

**Affiliations:** Department of Hematology, First Hospital of Shanxi Medical University, Taiyuan, Shanxi, China

**Keywords:** acute myeloid leukemia, elderly patients, post-chemotherapy myelosuppression, supportive therapy, umbilical cord blood infusion

## Abstract

Elderly AML (Acute Myeloid Leukemia) patients exhibit increased mortality owing to chemotherapy-induced myelosuppression. Umbilical cord blood (UCB) contains hematopoietic stem/progenitor cells and a spectrum of regenerative cytokines that actively promote hematopoietic regeneration. We investigated whether umbilical cord blood (UCB) infusion enhances hematopoietic recovery in elderly AML patients who experience chemotherapy-induced myelosuppression during post-chemotherapy supportive care.

**Methods:**

We retrospectively analyzed 95 elderly patients with AML in complete remission (CR1) who previously developed grade III–IV chemotherapy-induced myelosuppression. The control group (n=60) received standard supportive care, including granulocyte colony-stimulating factor (G-CSF), blood product transfusions, and antibiotic therapy. The experimental group (n = 35) underwent infusion of unrelated umbilical cord blood (UCB; CD34^+^ cells ≥2×10^6^/kg) within 24 hours post - chemotherapy, along with standard supportive care.

**Results:**

Neutrophil recovery duration (≥1.0×10^9^/L) was reduced in the UCB group (16 VS 23 days, P<0.01). Platelet recovery (≥50×10^9^/L) was also shorter (13 days vs. 19 days, P<0.01), though hemoglobin recovery (≥70 g/L) did not differ significantly (18 days vs. 21 days, P>0.05). The incidence of grade III–IV infections decreased to 31.43% (vs. 55.00%, P<0.05), that of bleeding events decreased to 11.43% (vs. 33.33%, P<0.05), that of transfusion-related allergic reactions decreased to 17.14% (vs. 26.67%, P<0.05), and chemotherapy delay/reduction rates decreased to 14.29% (vs. 35.00%, P<0.05). The overall survival (OS) was significantly longer in the UCB group, and the 2-year relapse-free survival (RFS) was higher (P<0.05). The most common adverse events were transfusion-related (e.g., rash and transient fever). Graft-versus-host disease (GVHD) was not observed in the UCB group.

These findings indicate that UCB infusion enhances hematopoietic recovery, reduces complications, and improves the survival of elderly patients with AML after chemotherapy, likely through the synergistic effects of CD34^+^ cells and cytokines.

## 1 Introduction

Acute Myeloid Leukemia (AML) is a prevalent hematologic malignancy whose incidence increases with age (1) Elderly patients with AML generally exhibit low remission rates and poor prognosis, with individuals over 60 years of age facing particularly unfavorable outcomes; the 5-year relative survival rate is only 12.5% (2).Current Chinese AML guidelines, alongside the WHO and ELN recommendations, advise that induction therapy for older adults should be tailored based on tolerance assessments, such as the Ferry score, permitting either standard regimens or reduced intensity/targeted therapies. Post-remission strategies primarily involve conventional cytotoxic chemotherapy (e.g., intermediate- or high-dose cytarabine) or allogeneic hematopoietic stem cell transplantation (allo-HSCT) (3).Although allo-HSCT offers the best potential for cure in AML (4), the majority of patients aged ≥60 are ineligible due to donor scarcity, patient preference, or biological constraints, leading to high relapse rates in this population (5).A critical limitation of chemotherapy and targeted agents is their ability to induce myelosuppression (6).This condition, which is characterized by neutropenia, thrombocytopenia, and anemia, is the most frequent and serious dose-limiting toxicity that significantly affects treatment feasibility and outcomes.

Myelosuppression is a major adverse effect of chemotherapy and is pathophysiologically characterized by depletion of the hematopoietic stem cell pool, damage to the bone marrow microenvironment, and delayed immune reconstitution. Clinically, it manifests as infections, hemorrhage, and treatment delays or dose reductions, significantly compromising therapeutic efficacy and the patient’s quality of life. Notably, elderly patients (≥70 years) exhibit a particularly poor 2-year survival rate of only 40% (7, 8). Current management primarily relies on granulocyte colony-stimulating factor (G-CSF) and the transfusion of platelets or red blood cells. However, these supportive measures are limited by slow efficacy (median time to neutrophil recovery is 18.64 days (9), and platelet recovery requires 19.71 days) (10, 11), high rates of severe complications (infection rate 39.3% (12), bleeding risk 21.2% (13)), donor dependency (for allogeneic transfusion), and immunologic reactions. Although allogeneic hematopoietic stem cell transplantation can restore bone marrow function, its applicability is restricted to select patients and carries significant risks, especially in elderly populations (14).

Umbilical cord blood (UCB) is rich in hematopoietic stem cells (HSCs), hematopoietic progenitor cells (HSPCs), and a variety of cytokines (e.g., IL-6 and SCF), positioning it as a critical cellular resource for hematopoietic reconstitution following myelosuppression. Preliminary safety and efficacy studies were performed in patients with advanced myeloid malignancies. Umbilical cord blood transplantation (UCBT) has been successfully employed in the treatment of hematologic malignancies such as chemotherapy-sensitive lymphomas and acute leukemia (15). Compared to bone marrow and peripheral blood stem cells, UCB offers several advantages, including lower immunogenicity (relatively flexible HLA matching requirements), strong proliferative capacity, convenient collection procedures, and the absence of ethical controversies (16, 17). Previous studies have suggested that UCB may promote bone marrow recovery through direct supplementation with hematopoietic cells and/or the paracrine secretion of cytokines (18).

This retrospective clinical study aimed to evaluate the efficacy and safety of UCB infusion as an adjunctive consolidation therapy in elderly patients with AML, including the assessment of transfusion-related adverse reactions, infectious complications, and other outcomes; and to explore its potential mechanisms of action, thereby providing a basis for optimizing supportive care strategies for myelosuppression.

## 2 Materials and methods

### 2.1 Diagnostic criteria

Bone marrow cytology: proportion of bone marrow blasts ≥20% (morphological detection). Immunophenotyping: identification of leukemia-associated immunophenotypes (LAIP) specific to acute myeloid leukemia using flow cytometry. Minimal residual disease (MRD) detection: assessed by multiparameter flow cytometry. MRD negativity was defined as an MRD <0.01%. Samples containing 100,000 or more cells were considered eligible for evaluation, whereas samples with fewer than 100,000 cells were deemed non-eligible. Genetic testing included molecular genetic analysis and chromosomal karyotyping.

Response assessment: Complete remission (CR) was defined according to the 2022 European LeukemiaNet (ELN) recommendations and the 2022 WHO classification, in line with the current Chinese guidelines for adult AML (non-APL). CR was defined as bone marrow blasts <5% with no circulating blasts or blasts with Auer rods, absence of extramedullary leukemic involvement, and hematologic recovery with an absolute neutrophil count ≥1.0 × 10^9^/L and a platelet count ≥100 × 10^9^/L. Patients meeting the marrow and extramedullary criteria but with residual neutropenia (<1.0 × 10^9^/L) or thrombocytopenia (<100 × 10^9^/L) were classified as CR with incomplete hematologic recovery (CRi). Remission was confirmed by bone marrow morphology and multiparameter flow cytometry, and measurable residual disease (MRD) was assessed by multiparameter flow cytometry, with MRD negativity defined as <0.01%.

### 2.2 Inclusion criteria

Age ≥60 years. Diagnosed with AML (non-M3) according to the 2022 WHO criteria, having achieved complete remission after one or two cycles of induction chemotherapy, and currently in the consolidation therapy phase. Unsuitability-for or unwillingness to undergo allo-HSCT. History of grade III–IV myelosuppression following prior chemotherapy. ECOG performance status score of 0–2. Left ventricular ejection fraction (LVEF) ≥50% as measured by echocardiography. Normal range of urea and creatinine; alanine aminotransferase (ALT) ≤2.5 × upper limit of normal (ULN), aspartate aminotransferase ≤2.5 × ULN, total bilirubin ≤1.5 × ULN. Written informed consent was obtained from the patient or legal guardian.

### 2.3 Exclusion criteria

History of severe allergic reactions to blood product transfusions (e.g., anaphylactic shock). ECOG score >2. Impaired function of major organs meeting any of the following. Cardiac: LVEF <50% or NYHA class III–IV heart failure; Hepatic: Child–Pugh class B or C, or ALT/AST >3 × ULN; Renal: eGFR <30 mL/min/1.73 m^2^; Pulmonary: DLCO <40% of predicted value. Subjects with central nervous system (CNS) involvement. Uncontrolled bacterial, fungal, or viral infection (e.g., HIV, active hepatitis). Other concurrent active malignancies. Known intolerance or allergy to agents of the same class. Presence of uncontrolled or severe medical conditions. Inability to comply with follow-up or incomplete treatment records.

### 2.4 Intervention conditions

#### 2.4.1 Chemotherapy regimens:

The induction protocols consisted of IA, VA, and V+HAG. The IA regimen comprised idarubicin 8 mg/m^2^/d on days 1–3 and cytarabine 100 mg/m^2^/d on days 1-7. The VA regimen included venetoclax (100 mg on day 1, 200 mg on day 2, and 400 mg on days 3-28) and azacitidine 75 mg/m^2^/d on days 1-7. The V+HAG regimen consisted of venetoclax (at the same dosage as in the VA regimen), homoharringtonine 1.4 mg/m^2^ (maximum 2 mg/d) on days 1-10, cytarabine 10 mg/m^2^ every 12 hours on days 1-14, and granulocyte colony-stimulating factor (G-CSF, 200 µg/m^2^/d on days 1–14 if white blood cell count <10×10^9^/L).

### 2.5 Experimental grouping and intervention measures

#### 2.5.1 Group design

This was a retrospective, non-randomized cohort study, and no randomization procedure was applied. All patients fulfilled the same predefined inclusion and exclusion criteria (Sections 2.2 and 2.3). The experimental group received conventional supportive therapy plus unrelated umbilical cord blood (UCB) infusion, whereas the control group received conventional supportive therapy alone. Allocation to the UCB group was determined by (1) the availability of an unrelated UCB unit meeting the predefined quality criteria (CD34^+^ ≥ 2×10^6^/kg, pathogen-negative) (2); the treating physician’s assessment of suitability for infusion based on organ function and the absence of contraindications; and (3) the patient’s informed consent and willingness to undergo the additional, self-funded UCB infusion. Patients for whom no qualifying UCB unit was available, or who declined the infusion, received standard supportive care alone and constituted the control group.

#### 2.5.2 UCB-related requirements and procedures

The source of UCB: Obtained from Shandong Cord Blood Bank (SINOCORD). Pretreatment Process: Thawed and resuscitated prior to infusion; irradiated (dose, 25–30 Gy) to prevent GVHD. Transportation Method: Transported entirely with dry ice for preservation. Quality Standards for UCB: Single UCB unit CD34^+^ cell count ≥2×10^6^/kg; Pathogen testing negative.

#### 2.5.3 Infusion procedure

Timing: Initiated within 36 h of chemotherapy. Dosage: Each patient received 2 units (total volume 200–480 ml). Premedication: Intravenous dexamethasone (5 mg) and calcium gluconate (10 ml) were administered prior to the infusion to prevent transfusion-related allergic reactions. Monitoring involved continuous ECG monitoring during infusion.

#### 2.5.4 Conventional supportive therapy (applied to both groups)

Both groups received supportive care during the myelosuppression phase after chemotherapy, including: G-CSF (initiated when neutrophil count <1.0×10^9^/L); intravenous thrombopoietin (TPO); platelet transfusion when platelet count <10×10^9^/L or bleeding tendency occurred; red blood cell transfusion when hemoglobin <70 g/L; and necessary anti-infective and hemostatic supportive treatments.

### 2.6 Primary outcome measures

Hematopoietic recovery time:​time to neutrophil recovery (absolute neutrophil count ≥1.0×10^9^/L), platelet recovery (platelet count ≥50×10^9^/L), and hemoglobin recovery (Hb ≥70 g/L). Complication rates: clinical bleeding events (mucocutaneous or visceral hemorrhage occurring within 72 hours of visit); clinical infection events (fever, respiratory or gastrointestinal infections); and chemotherapy delay/reduction rate (proportion of treatment plan adjustments due to myelosuppression). Overall survival (OS) was defined as the time from diagnosis to death from any cause or the last follow-up, and relapse-free survival (RFS) was defined as the time from complete remission to disease relapse or death from any cause. Assessment Timing: Therapeutic effects were evaluated after each consolidation cycle. Follow-up assessments were scheduled monthly in the first year, and quarterly thereafter.

### 2.7 Criteria for treatment-related adverse event assessment

Treatment-related adverse events (including hematological and non-hematological events) were defined as adverse reactions occurring after treatment initiation. Severity was graded according to the National Cancer Institute’s Common Terminology Criteria for Adverse Events (CTCAE v5.0): grades 1-2, mild events; and grades 3-4, severe events. Adverse events were monitored at the initiation of treatment and continued until the final follow-up.

### 2.8 Data collection and analytical methods

This retrospective study collected data using the following approaches: baseline characteristics (including genetic mutation profiles, chemotherapy regimens, and prior treatment history) were obtained from the clinical medical record system; survival outcomes and complication data were acquired during outpatient visits and telephone follow-ups; and follow-up was conducted at outpatient clinics or by telephone every 3–6 months, with the final follow-up date set at October 30, 2025.

### 2.9 Statistical analysis

Statistical analyses were performed using SPSS 25.0, and graphs were generated using GraphPad Prism 9.30. Categorical variables are presented as “n (%)” and compared using the chi-square test or Fisher’s exact test. Normally distributed continuous variables were expressed as “mean ± standard deviation” and compared between groups using independent samples t-tests. Within-group comparisons were performed using paired samples t-tests. Non-normally distributed continuous variables are reported as medians (interquartile range) “M (P25, P75)” and compared using the Mann–Whitney U test. Survival rates were estimated using the Kaplan–Meier method and compared using the log-rank test. A two-sided α level of 0.05 was used for all tests, with P < 0.05 considered statistically significant.

## 3 Results

### 3.1 Baseline characteristics

This retrospective study analyzed the clinical data of 95 adult patients with newly diagnosed acute myeloid leukemia (excluding acute promyelocytic leukemia, APL) treated at our institution between January 2019 and June 2025. The study protocol was approved by the Institutional Ethics Committee (Approval No. 2023-026), and all procedures complied with the ethical standards established by the review board. Written informed consent was obtained from all participants (**Table 1**).

**Table 1:** Baseline data of patients in two groups.

Item	Experimental group (n=35)	Control group (n=60)	χ^2^	P
**Gender**			0.076	0.783
Female	13 (37.14)	24 (40.00)		
Male	22 (62.86)	36 (60.00)		
**Age (mean ± SD)**	76.57 ± 9.48	70.85 ± 7.60	3.225	0.002
**ECOG Score**			0.291	0.865
0	5 (14.29)	11 (18.33)		
1	11 (31.43)	17 (28.33)		
2	19 (54.29)	32 (53.33)		
**ELN 2022 Risk Classification**			0.180	0.914
Intermediate	17 (48.57)	31 (51.67)		
Favorable	11 (31.43)	19 (31.67)		
Unfavorable	7 (20.00)	10 (16.67)		
**Karyotype**			1.925	0.964
inv(16)	0 (0.00)	1 (1.67)		
t(8;21)	3 (8.57)	5 (8.33)		
Normal Karyotype	24 (68.57)	41 (68.33)		
Complex Karyotype	5 (14.29)	6 (10.00)		
Others	2 (5.71)	4 (6.67)		
Gene Mutation Type			2.586	0.99
CEBPA	10 (28.57)	13 (21.67)		
NPM1	8 (22.86)	12 (20.00)		
FLT3-ITD	3 (8.57)	4 (6.67)		
DNMT3A	4 (11.43)	7 (11.67)		
Others	10 (28.57)	24 (40.00)		

ECOG, Eastern Cooperative Oncology Group Performance Status Scale; ELN 2022 Risk Classification, Acute Myeloid Leukemia Risk Stratification System. Bold values indicate statistically significant differences between groups (P < 0.05).

Of the 95 patients, 58 were male and 37 were female, with an overall median age of 73.02 years. The UCB and control groups were comparable with respect to sex, ECOG performance status, ELN 2022 risk stratification, karyotype, and gene mutation profiles. Patients in the UCB group were older than those in the control group; the implications of this difference for the survival analysis are addressed in the Discussion.

All patients met the 2022 WHO diagnostic criteria for AML, and none had a history of myelodysplastic syndrome (MDS) transformation. According to the 2022 ELN risk classification, the proportion of patients in the favorable-risk category was 31.43% (11/35) in the experimental group and 31.67% (19/60) in the control group. The intermediate-risk category accounted for 48.57% (17/35) and 51.67% (31/60) of the experimental and control groups, respectively; and the adverse-risk comprised 20.00% (7/35) and 16.67% (10/60) of the two groups respectively. Cytogenetic analysis revealed complex karyotypes in 14.29% (5/35) and 10.00% (6/60) of the experimental and control groups, respectively. The predominant gene mutations were CEBPA (28.57% experimental vs. 21.67% control), NPM1 (22.86% vs. 20.00%), and DNMT3A (11.43% vs. 11.67%).

### 3.2 Treatment response

All patients achieved complete remission (CR1) after 1–2 cycles of induction therapy. By the final follow-up date (June 30, 2025), all patients had completed at least six months of follow-up or reached the primary study endpoint.

Induction regimens included venetoclax + azacitidine (VA; 82.86% in the experimental group vs. 80.00% in the control group), idarubicin + cytarabine (IA; 14.29% vs. 13.33%), and venetoclax + homoharringtonine + cytarabine + G-CSF (V-HAG; 2.86% vs. 6.67%). No significant difference in the minimal residual disease (MRD) status was observed before and after consolidation therapy.

Hematopoietic recovery was significantly accelerated in the experimental group: time to neutrophil recovery (≥1.0×10^9^/L) was 16.0 days in this group compared to 23.0 days in the control group; and platelet recovery (≥50×10^9^/L) was 13.0 days compared to 19.0 days in the control group. Hemoglobin recovery (≥70 g/L) was shorter in the experimental group (18.0 days vs. 21.0 days) but not a statistically significant level. The mean duration of myelosuppression was shorter in the experimental group (15.20 days) compared to the control group (17.40 days), and the average hospital stay was also shorter (21.51 days) than that observed in the control group (23.50 days) (**Table 2**).

**Table 2:** Comparison of peripheral blood cell recovery times during Post-chemotherapy myelosuppressive phase.

Item	Experimental group (n=35)	Control group (n=60)	*P*
Recovery Time of Absolute Neutrophil Count (days)	16.0 (13.0, 23.0)	23.0 (14.0, 38.8)	0.006
Recovery Time of Platelet Count (>50×10^9^/L, days)	13.0 (12.0, 32.0)	19.0 (15.0, 42.8)	0.024
Recovery Time of Hemoglobin (>70g/L, days)	18.0 (14.0, 26.0)	21.0 (16.0, 34.8)	0.057

**Figure 1:**
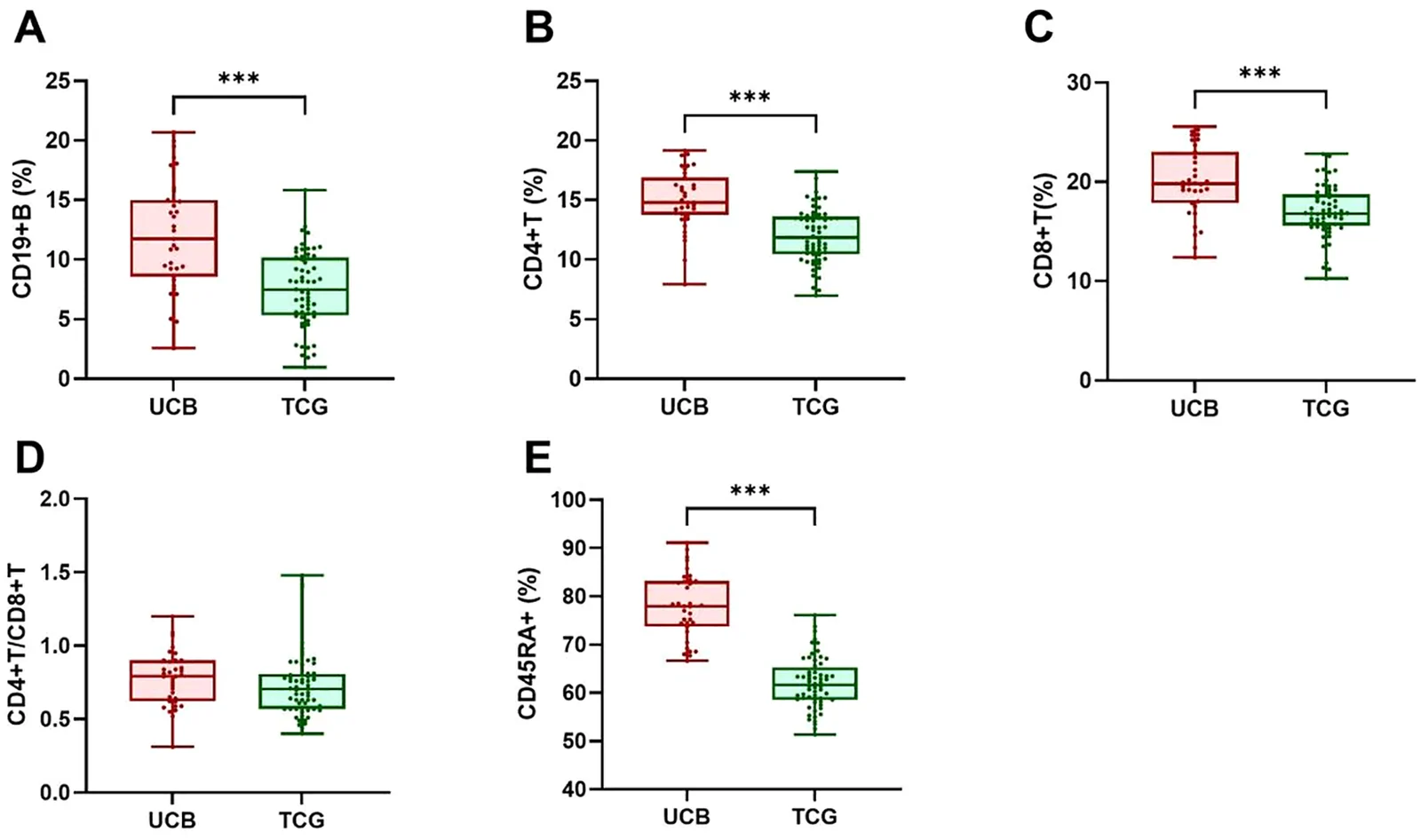
Comparison of peripheral blood lymphocyte recovery times (Day 14). **(A)** CD19+B Cell Proportion on Day 14 (%). **(B)** CD4+T Cell Proportion (%). **(C)** CD8+T Cell Proportion (%). **(D)** CD4+T/CD8+T Ratio. **(E)** CD45RA+ Cell Proportion (%). Asterisks indicate statistical significance, with *** indicating P < 0.001. UCB, Experimental group with unrelated cord blood infusion; TCG, Control group with traditional consolidation therapy.

By day 14, lymphocyte recovery in the experimental group showed a significant increase in B cells (12.1%) compared to the control group (7.5%, P < 0.05), elevated CD4^+^ T cells (15% and 12% in the experimental and control groups, respectively), an improved CD4^+^/CD8^+^ ratio (0.77 and 0.73 in the experimental and control groups, respectively), and a higher proportion of naïve T cells (CD45RA^+^; 78% and 62% in the experimental and control groups, respectively).(**Figure 1**).

At the follow-up endpoint, 29 of the 35 patients (82.9%) in the experimental group survived. Among the six deaths, three were due to disease progression, two resulted from treatment discontinuation for economic reasons, and 1 was attributed to a secondary malignancy.

### 3.3 Safety profile

All enrolled patients were included in treatment-related toxicity analysis. Treatment-emergent adverse events during the post-remission consolidation phase, categorized by type and severity (**Table 3**).

**Table 3:** Comparison of incidence of complications between two groups.

Item	Experimental group (n=35)	Control group (n=60)	P
Duration of Myelosuppression (days)	15.20 ± 3.49	17.40 ± 4.52	0.015
Incidence of Complications, n (%)			
Infection	11 (31.43)	33 (55.00)	0.026
Bleeding	4 (11.43)	20 (33.33)	0.018
Transfusion Allergic Reaction	6 (17.14)	16 (26.67)	0.031
Rate of Chemotherapy Delay/Reduction	5 (14.29)	21 (35.00)	0.029
Length of Hospital Stay (days)	21.51 ± 3.78	23.50 ± 4.60	0.033

The incidence of infection was significantly lower in the experimental group (31.43%, 11/35) than in the control group (55.00%, 33/60). Bleeding events were less frequent in the experimental group (11.43%, 4/35) than in the control group (33.33%, 20/60). The rate of transfusion-associated allergic reactions was 17.14% in the experimental group and 26.67% in the control group, whereas the incidence of overall hypersensitivity reactions was higher in the experimental group (25.71%, 9/35) than in the control group (8.33%, 5/60). The chemotherapy delay or dose reduction rate was significantly lower in the experimental group (14.29%, 5/35) than that in the control group (35.00%, 21/60).

Gastrointestinal adverse reactions were exclusively grades 1–2, with no grade 3–4 events reported in either group. The incidence was 20.00% (7/35) and 15.00% (9/60) in the experimental and control groups, respectively, with no statistically significant differences. Hepatic impairment events were also limited to grades 1–2, occurring in 2.86% (1/35) of participants the experimental group and 18.33% (11/60) of participants in the control group. No cases of mucositis, dermatological disorders, or sepsis were observed in either group. Importantly, no clinical manifestations of graft-versus-host disease (GVHD) were observed in any study participant.

### 3.4 Survival analysis

**Figure 2:**
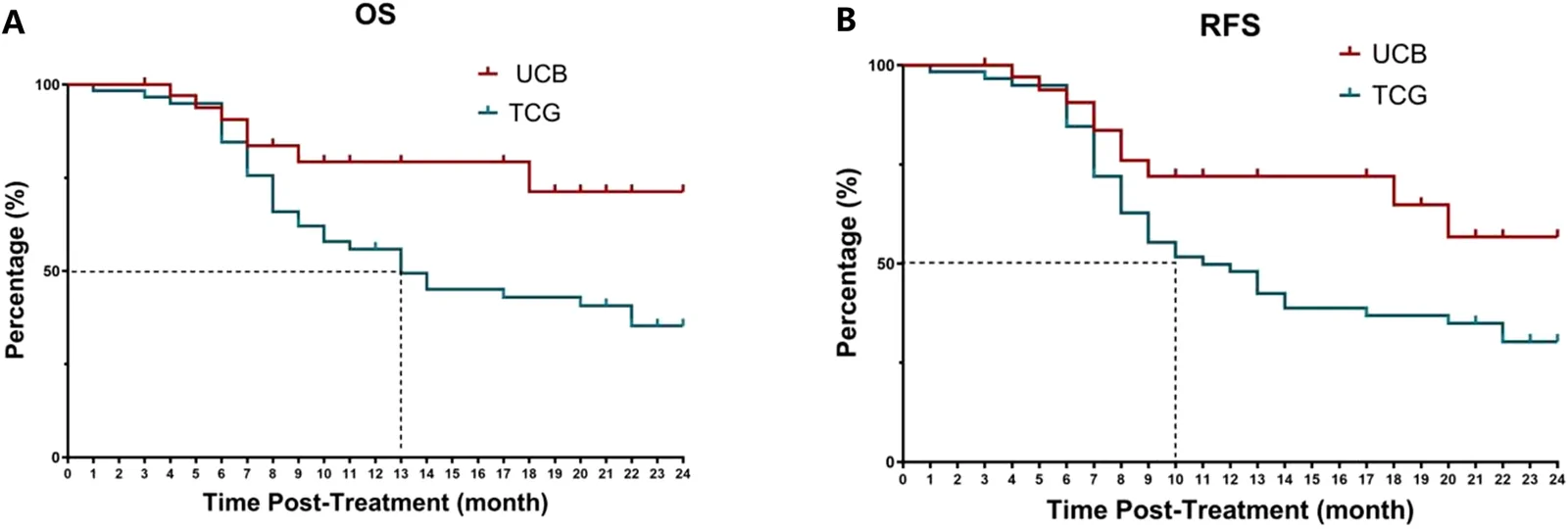
Survival curves of patients in two groups. **(A)** Overall Survival Curve (OS: Overall Survival). **(B)** Relapse-Free Survival Curve (RFS: Relapse-Free Survival). UCB, Experimental group with unrelated cord blood transfusion; TCG, Control group with traditional consolidation therapy.

Survival outcomes over the 24-month observation period are shown in **Figure 2** and favored the UCB group for both overall survival (OS) and relapse-free survival (RFS). Overall survival. The Kaplan-Meier OS curves were superimposable during the first months after treatment and separated progressively from approximately month 6, after which they remained clearly apart (**Figure 2A**). Median OS was 13 months in the TCG group, whereas median OS was not reached in the UCB group, in which 6 deaths occurred during follow-up. The estimated OS rate was approximately 80% in the UCB group versus 55% in the TCG group at 12 months, and approximately 72% versus 35% at 24 months. The difference in OS between the two groups was statistically significant (log-rank *P* < 0.05).

Relapse-free survival. The RFS curves diverged progressively during follow-up, with the UCB group maintaining a higher relapse-free probability throughout the observation period (**Figure 2B**). Median RFS was 10 months in the TCG group and was not reached in the UCB group. The estimated RFS rate was approximately 73% in the UCB group versus 45% in the TCG group at 12 months, and approximately 57% versus 31% at 24 months. Relapse and death events occurred earlier and more frequently in the TCG group, and the difference in RFS was statistically significant (log-rank *P* < 0.05).

Taken together, these findings indicate that, in this cohort, UCB infusion was associated with longer OS and RFS in elderly AML patients with chemotherapy-induced myelosuppression.

## 4 Discussion

The incidence of acute myeloid leukemia (AML) in the elderly continues to increase despite considerable progress in supportive care and targeted therapeutic approaches. Novel agents, particularly venetoclax-based regimens combined with hypomethylating agents for induction therapy (19), as well as advances in supportive management, have expanded the treatment options and increased the likelihood of remission. However, older patients frequently exhibit delayed hematopoietic recovery following chemotherapy compared to younger cohorts. This impairment stems from an age-related decline in bone marrow function, as evidenced by the extended median recovery times for neutrophils (29 days) (20) and platelets (25 days) (21). Data from the control group in our study are consistent with these findings, showing median neutrophil and platelet recovery times of 18.64 days and 19.71 days, respectively, among older AML patients after achieving first complete remission (CR1). During the myelosuppressive phase, the incidence of grade III–IV infections reached 38.3%, accompanied by a significant increase in hemorrhagic risk. These complications contribute to higher treatment-related mortality and often necessitate dose reduction or chemotherapy delay (22) due to poor tolerance, thereby increasing the risk of disease relapse.

Umbilical cord blood (UCB) is a valuable cellular resource for enhancing hematopoietic recovery owing to its diverse composition of stem cells, cytokines, and other biologically active components. Its efficacy has been established in allogeneic and posttransplant reconstitution settings (23). UCB is notably rich in CD34^+^ cells (reaching concentrations of 3.76×10^6^/kg), which exhibit a proliferative capacity 3.2-fold greater than that of bone marrow-derived cells (24). Moreover, UCB contains various hematopoietic cytokines, such as IL-6 and G-CSF, which contribute to the reconstitution of the hematopoietic cell pool and restoration of the bone marrow microenvironment. Another advantage is the relative flexibility in HLA matching; four out of six loci are sufficient for compatibility (25), which results in reduced immunogenicity and a lower incidence of graft-versus-host disease (GVHD) (0.8%) (26), underscoring its favorable safety profile. Supporting these benefits, a study involving 23 AML patients aged ≥60 years in first complete remission (CR1) demonstrated that treatment with decitabine plus intermediate-dose cytarabine, together with infusion of HLA-mismatched G-CSF-mobilized peripheral blood stem cells (the D-GPBSC regimen), shortened median platelet (≥20×10^9^/L) and neutrophil recovery times to 14 and 12 days, respectively. The same study reported a 2-year overall survival (OS) of 55.4% and event-free survival (EFS) of 51% (27), reinforcing the potential of UCB-derived strategies to accelerate hematopoietic recovery.

Our results indicated that the neutrophil recovery time in the experimental group was 16 days, platelet recovery time was 13 days, and hemoglobin recovery time was 18.0 days. The incidence of severe complications such as infections and bleeding during the recovery period was reduced. In terms of efficacy, the experimental group not only exhibited faster hematopoietic recovery but was also associated with longer survival, with a median OS that was not reached versus 13 months in the control group and an estimated 24-month OS of approximately 72% versus 35%. The median RFS was not reached in the UCB group, with an estimated 24-month RFS of approximately 57% versus 31% in the control group, indicating more durable disease control.

At the immunological level, patients often experience poor recovery in the proportion and function of B lymphocytes (CD19^+^) and CD4^+^ T cells after chemotherapy-induced myelosuppression (28), increasing the risk of opportunistic infections. Umbilical cord blood promotes immune reconstitution with fewer transplantation-related complications. This is attributed to the unique immune tolerance properties of umbilical cord blood, where T cells display a predominantly naïve phenotype (29), differing from the predominance of central and effector memory T cells in adult peripheral blood. This immune characteristic helps to reduce post-transplant immune complications (30). Monitoring of lymphocyte subsets in this study showed that after umbilical cord blood infusion, immune recovery was faster in the experimental group than in the control group, with the proportion of CD19^+^ B cells reaching 12.1% and naïve T cells (CD45RA^+^) reaching 78% on day 14, suggesting that umbilical cord blood infusion may reduce the risk of infection; any potential effect on minimal residual disease and relapse risk was not demonstrated in our cohort and remains speculative. Natural killer (NK) cells derived from umbilical cord blood have been confirmed to possess key characteristics distinct from those of other sources and show potential for application in cancer treatment (31).

The favorable tolerability of umbilical cord blood (UCB) infusion makes it particularly suitable for elderly patients with multiple comorbidities and a fragile constitution. A significant reduction in the incidence of severe complications such as infections has been observed (32), contributing to an improved quality of life. In this study, no UCB infusion-related adverse reactions were observed. In contrast, patients in the control group, who did not receive UCB, experienced longer durations of myelosuppression and required more frequent transfusions, resulting in a higher incidence of transfusion-associated allergic reactions.

As these mechanisms were not directly evaluated in this study, the following interpretations are exploratory and hypothesis-generating. The ability of UCB to promote hematopoietic recovery may be attributed to the direct homing of UCB-derived hematopoietic stem cells (HSCs) to the bone marrow and the secretion of hematopoietic cytokines such as SCF and IL-6, which activate the endogenous hematopoietic microenvironment (33). Specific mechanisms include efficient homing of CD34^+^ hematopoietic stem cells from UCB to the bone marrow microenvironment via the CXCR4/SDF-1 axis (34), synergistic activation of host stromal cell function by multiple hematopoietic cytokines, accelerated immune reconstruction, and the unique immunomodulatory properties of UCB, which enhance the host anti-infective capacity while effectively reducing the risk of graft-versus-host disease.

This study has several limitations. First, its retrospective, single-center, non-randomized design may introduce selection bias, as allocation to UCB infusion was based on clinical suitability, availability of UCB units, and patient consent rather than randomization. Second, patients in the UCB group were significantly older than those in the control group; because older age is an adverse prognostic factor in AML, this imbalance would be expected to disadvantage rather than favor the UCB group, but residual unmeasured confounding cannot be fully excluded. Third, the sample size was relatively small, and no prospective sample size or statistical power calculation was performed, because the study population was determined by the number of eligible patients available during the study period; some endpoints may therefore have been underpowered, and the results should be interpreted with caution. These findings should be regarded as hypothesis-generating and require validation in larger, multicenter prospective studies, ideally randomized controlled trials, with formal power calculations and multivariable adjustment.

In summary, the findings of this study demonstrate that umbilical cord blood (UCB) infusion as an adjunctive therapy for chemotherapy-induced myelosuppression in elderly patients with AML safely and effectively accelerates neutrophil and platelet recovery, reduces complication risks, and improves treatment continuity. These results support the favorable clinical value and safety profile of UCB infusion. Further multicenter randomized controlled trials are warranted to optimize UCB dosing and timing. Single-cell sequencing technologies should be employed to elucidate the mechanisms of specific stem cell subpopulations in UCB, thereby advancing precision medicine for the management of AML in elderly patients.

## 5 Conclusion

This study confirmed that adjunctive umbilical cord blood infusion during the myelosuppressive phase following chemotherapy in elderly acute myeloid leukemia patients with AML significantly shortens neutrophil and platelet recovery times, decreases the incidence of infections and bleeding events, and reduces treatment delays. The regimen exhibited a favorable safety profile, with no severe graft-versus-host disease observed, thus providing a new clinical option for supportive care after chemotherapy.

## Data Availability

The raw data supporting the conclusions of this article will be made available by the authors, without undue reservation.
